# Current Status of the Use of Multifunctional Enzymes as Anti-Cancer Drug Targets

**DOI:** 10.3390/pharmaceutics14010010

**Published:** 2021-12-21

**Authors:** Carla S. S. Teixeira, Sérgio F. Sousa

**Affiliations:** 1 Associate Laboratory i4HB, Faculty of Medicine, Institute for Health and Bioeconomy, University of Porto, 4050-313 Porto, Portugal; carla.s.silva.teixeira@gmail.com; 2 UCIBIO—Applied Molecular Biosciences Unit, BioSIM—Department of Biomedicine, Faculty of Medicine, University of Porto, 4051-401 Porto, Portugal

**Keywords:** cancer, molecular-targeted therapies, multifunctional enzymes

## Abstract

Fighting cancer is one of the major challenges of the 21st century. Among recently proposed treatments, molecular-targeted therapies are attracting particular attention. The potential targets of such therapies include a group of enzymes that possess the capability to catalyze at least two different reactions, so-called multifunctional enzymes. The features of such enzymes can be used to good advantage in the development of potent selective inhibitors. This review discusses the potential of multifunctional enzymes as anti-cancer drug targets along with the current status of research into four enzymes which by their inhibition have already demonstrated promising anti-cancer effects in vivo, in vitro, or both. These are PFK-2/FBPase-2 (involved in glucose homeostasis), ATIC (involved in purine biosynthesis), LTA_4_H (involved in the inflammation process) and Jmjd6 (involved in histone and non-histone posttranslational modifications). Currently, only LTA_4_H and PFK-2/FBPase-2 have inhibitors in active clinical development. However, there are several studies proposing potential inhibitors targeting these four enzymes that, when used alone or in association with other drugs, may provide new alternatives for preventing cancer cell growth and proliferation and increasing the life expectancy of patients.

## 1. Introduction

Cancer has become one of the greatest barriers to the increase of life expectancy in almost every country in the world, and is one of the biggest medical challenges of the 21st century. According to GLOBOCAN, in 2020 there were a record 19.3 million new cases and 10 million deaths caused by cancer worldwide [1]. Cancer is a multifactorial disease developed when normal cells acquire mutations or alterations that provide them a growth and/or survival advantage, enabling them to multiply and form a tumor. The extraordinary capability of cancer cells to divide and proliferate is highly associated with their metabolic reprogramming [2,3].

One of the biggest challenges in cancer research is the complexity of cancer’s genomic landscape [4], which results in an enormous heterogeneity: there are more than 200 different recorded types of cancers, affecting as many as 60 human organs [5]. Over the years, several different approaches to fighting cancer have emerged including surgery, hormone therapy, radiation therapy, immunotherapy, chemotherapy, and more recently molecular-targeted therapy (reviewed in [6]). Although there have been significative advances in cancer survival, there are still many issues associated with the existing cancer therapies, including the existence of severe side effects, the acquisition of multidrug resistance, relapse, or even the possibility of developing new cancers caused by the treatment.

In recent years, molecular-targeted therapies have been gaining particular attention. Unlike the standard chemotherapies, which are cytotoxic (killing the cancer cells) and act on both rapidly dividing normal and on cancerous cells, molecular-targeted drugs are cytostatic (stopping cancer cell growth and/or proliferation) and only inhibit specific molecular targets that are involved in the growth and spread of cancer cells [7]. Therefore, molecular-targeted drugs are less prone to causing secondary effects [7]. Among the potential anti-cancer drug targets are the proteins abnormally expressed in some tumors. To date, a panoply of proteins have been identified as potential targets, including some enzymes that belong to special class generally designated as “multifunctional enzymes”. These enzymes share a common characteristic: they possess the capability to catalyze at least two different reactions in the same or in different overlapping or distant active sites.

In this review, we focus on four multifunctional enzymes that are abnormally expressed in different cancer cells, and that have been previously proposed as good drug targets for cancer treatment.

First, 6-phosphofructo-2-kinase (PFK-2)/fructose-2,6-bisphosphatase (FBPase-2) is involved in glucose homeostasis and encompasses two independent domains in the same monomer, each possessing an independent active site.

Second, 5-aminoimidazole-4-carboxamide ribonucleotide formyltransferase/inosine monophosphate cyclohydrolase (ATIC) is involved in purine biosynthesis and also encompasses two independent domains in the same monomer, each possessing an independent active site.

Third, Leukotriene A_4_ hydrolase (LTA_4_H) is involved in the inflammation process; its distinct catalytic activities occur in two distinct but overlapping active sites.

Fourth, Jumonji domain-containing protein 6 (Jmjd6) is involved in histone and non-histone posttranslational modifications, and uses the same active site to catalyze at least two different reactions.

In general, multifunctional enzymes are promising drug targets because they can be inhibited in multiple ways depending on their characteristics.

The most specific type of enzymatic inhibition relies on the use of inhibitors that are transition state analogs. The design of these inhibitors is based on the structure of the transition state of the rate-limiting step of a catalytic reaction, and their development therefore depends on a deep knowledge of the enzyme’s mechanism that can only be accomplished by combining experimental and computational data [8]. However, the design of such inhibitors is not always possible, either because there is no atomistic description of the transition state structures or because the molecules obtained cannot be used as therapeutical drugs due to their pharmacokinetic properties or toxicity. The choice of multifunctional enzymes as drugs targets is advantageous because their complexity enables inhibition using multiple different approaches.

We start this review with a global description of the biological role of each enzyme, followed by a description of its tertiary and quaternary structure; we then provide evidence about its role in cancer development and/or survival; finally, we describe the most promising inhibitors proposed to date targeting each enzyme.

## 2. 6-phosphofructo-2-kinase/fructose 2,6-bisphosphatase (PFK-2/FBPase-2)

### 2.1. Biological Role

Fructose 2,6-bisphosphate (Fru-2,6-P_2_) is an important signal molecule that can be found in all mammalian tissues [9,10,11]. In the liver, Fru-2,6-P_2_ plays an important role in the control of glucose homoeostasis by allowing the liver to switch from glycolysis to gluconeogenesis through the inhibition of FBPase-1 (fructose-1,6-bisphosphatase), a regulatory enzyme of gluconeogenesis [12]. When mammals are experiencing fasting conditions, the α-cells of the pancreas secrete a linear peptide hormone called glucagon, which decreases the concentration of hepatic Fru-2,6-P_2_ and thereby relieves the inhibition of FBPase-1, allowing gluconeogenesis to prevail [9]. In most mammalian tissues, which do not contain FBPase-1, Fru-2,6-P_2_ acts as a potent positive allosteric effector of 6-phosphofructo-1-kinase (PFK-1), an enzyme that catalyzes one of the most critical steps of glycolysis—the conversion of fructose 6-phosphate (Fru-6-P) and ATP to fructose 1,6-bisphosphate and ADP [9,11,13,14].The levels of Fru-2,6-P_2_ are controlled by a family of bifunctional enzymes that possess in the same peptide a 6-phosphofructo-2-kinase (PFK-2) and a fructose-2,6-bisphosphatase (FBPase-2) domain.

**Figure 1:**
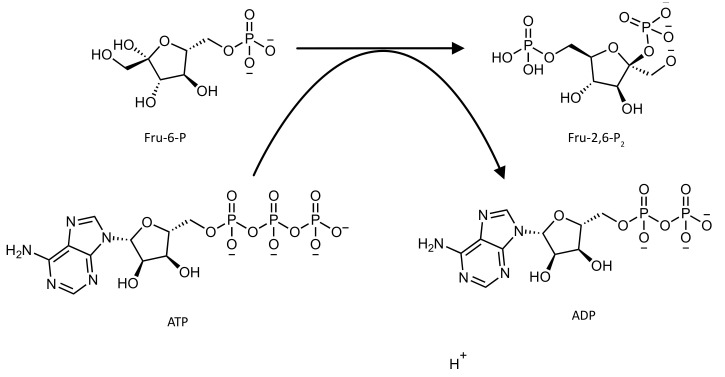
Schematic representation of the reaction catalyzed by the PFK-2 domain of the PFK-2/FBPase-2 enzyme.

The PFK-2 domain (E.C. 2.7.1.105) synthesizes Fru-2,6-P_2_ from frutose-6-phosphate (Fru-6-P) and ATP (Figure 1).

**Figure 2:**
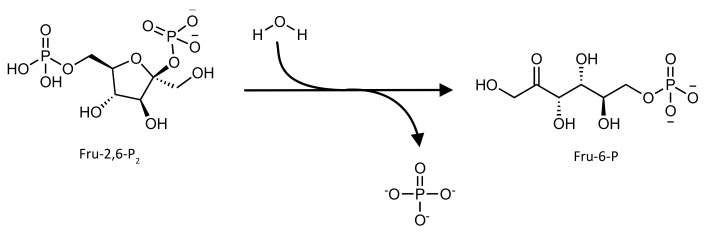
Schematic representation of the reaction catalyzed by the FBPase-2 domain of the PFK-2/FBPase-2 enzyme.

The FBPase-2 domain (E.C. 3.1.3.46) hydrolyzes Fru-2,6-P_2_ into Fru-6-P and inorganic phosphate (Figure 2) [15,16].

The balance between the activity of the two catalytic domains ultimately determines the concentration of Fru-2,6-P_2_. This important regulatory function of Fru-2,6-P_2_ in carbohydrate metabolism requires tight regulation of its concentration as a function of the cell needs. This is accomplished by the existence of different PFK-2/FBPase-2 isoenzymes [17] with different kinetic and regulatory mechanisms, which regulate the glycolysis and gluconeogenesis pathways in different tissues under various physiological conditions [18].

Mammals express four PFK-2/FBPase-2 isoenzymes, which are encoded by four different genes, *PFKFB1* to *PFKFB4* [19]. Although the different isoenzymes were initially named according to the tissue from which they were first purified (PFKFB1 in the liver, PFKFB2 in the heart, PFKFB3, in the brain and placenta, and PFKFB4 in the testes), more recent evidence has demonstrated that they are expressed in other tissues as well, and they are now classified according to their coding gene [9]. Each isozyme has several isoforms that share the same catalytic core as the parent isoenzyme but differ in the flanking sequences. These variable sites in each isoform are subject to different post-translational modifications, usually phosphorylation by different protein kinases, that modulate the relative activities of their catalytic domains under the control of cellular signaling pathways. This complex regulation allows the cell to adapt the carbohydrate metabolism in response to extracellular stimuli (e.g., hormones, growth factors, nutritional state) [18].

### 2.2. Protein Structure

The different PFK-2/FBPase-2 isoenzymes differ in the sequence of their bifunctional catalytic core, while their various isoforms conserve the catalytic core of the parent isoenzyme but differ in their N- and C-terminal ends where the post translation modifications take place. Although those sequence variations result in relevant conformational differences among the different proteins, their overall 3D structure is quite similar.

**Figure 3:**
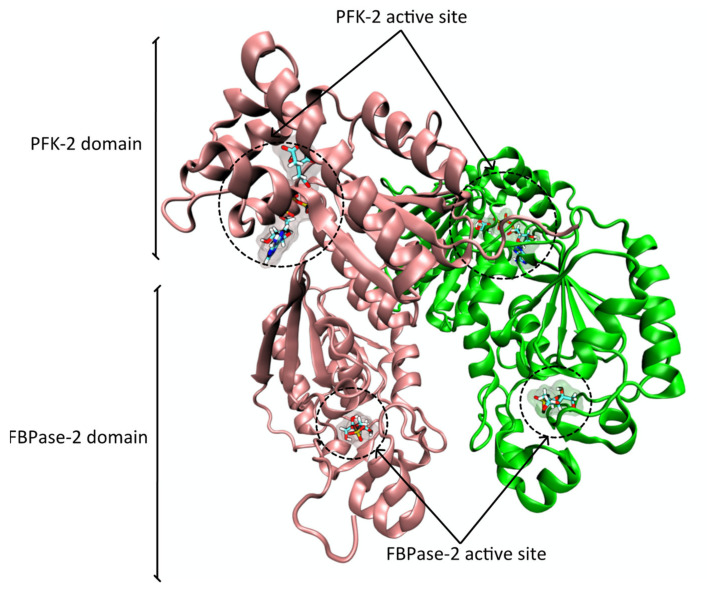
Ribbon representation of the human PFK-2/FBPase-2 (PFKFB2) enzyme with the PDB ID 5HTK [26], obtained with VMD. The PFK-2 active site harbors an ATP and a citrate molecule (inside the Fru-6-P binding pocket) and the FBPase-2 active site harbors a frutose-6-phosphate molecule inside the F-2,6-P_2_ binding pocket. All ligands are represented in licorice.

The PFK-2/FBPase-2 is a homodimer composed of two 55-kDa monomers. Each monomer possesses a kinase domain (E.C. 2.7.1.105) at the N-terminal and a bisphosphatase domain at the C-terminal end of the protein (Figure 3) [9,16,20]. Observation of the global 3D structure of the homodimer shows that the PFK-2 domains come together in a head-to-head fashion while the FBPase-2 domains are almost independent, with few dimeric point interactions the number and nature of which (e.g., hydrogen bridges) vary among the different isoenzymes [9,16,20]. These observations are in line with experimental data showing that when expressed independently, the PFK-2 domain forms inactive aggregates [21], while the FBPase-2 domain retains it catalytic activity [22].

The differences in the binding pockets (where catalysis occurs) in the N- and C-terminal flanking sequences (where the post translation modifications take place) and in the dimeric interface contacts among the different isoenzymes and respective isoforms affects both the conformational stability and affinity for the substrate of Fru-2,6-P_2_, ultimately resulting in enzymes with different kinetics [9]. The kinase/phosphatase activity ratio is above 2.5/1 for PFKFB1 and PFKFB2, 730/1 for PFKFB3 and about 4.6/1 for PFKFB4 [23,24,25].

The advantage of expressing two independent catalytic domains encoded by a fused gene in the same monomer is the simplicity of both short-term control (by regulating the activity of the two domains trough post translation modifications and allosteric modulation) and long-term control (through the expression of two catalytic domains from a single mRNA molecule) [9].

### 2.3. Role in Cancer

To support their continuous growth and proliferation under challenging conditions, most cancer cells have a markedly modified energy metabolism in comparison with normal cells [27,28,29]. Both normal and cancer cells predominantly use glucose, the most abundant nutrient in the blood, to generate ATP [30]. The overexpression of several glycolytic genes in many tumors [27,28,29] allows them to change from respiration to a glycolytic phenotype even in aerobic conditions, a phenomenon known as the Warburg effect [31].

Several studies have shown that the Fru-2,6-P_2_ concentration is significantly higher in cancer cells with a glycolytic phenotype than in normal cells [32,33,34]. The increased levels of Fru-2,6-P_2_ allows the transformed cells to maintain a high glycolytic flux despite the presence of the PFK-1 inhibitor, ATP.

ATP inhibits PFK-1 activity as part of the negative feedback loop that limits glycolytic flux under aerobic conditions, the so-called Pasteur effect [35]. The presence of high levels of Fru-2,6-P_2_, which is a positive allosteric effector of PFK-1, relieves the ATP inhibition [14] and allows the cancer cells to maintain a high glycolytic flux. This is advantageous for the transformed cells because the maintenance a high glycolytic flux allows them to produce higher ATP rates when compared with oxidative phosphorylation, and provides them with intermediates that are vital for other important biosynthetic pathways, (for example, ribose sugars for nucleotide synthesis; hexose sugar derivatives, glycerol and citrate for lipid synthesis; non-essential amino acids and NADPH, which are important for nucleotide and fatty acid biosynthesis and for the maintenance of cellular redox balance [18].

Due to their role in the modulation of Fru-2,6-P_2_ levels, the PFK-2/FBPase-2 enzymes have been pointed out as key players in the glycolytic phenotype of cancer cells and consequently in the regulation of these cells’ metabolic activity [18]. This observation is supported by the fact that PFK-2/FBPase-2 mRNAs are overexpressed in human lung cancers [36] and by the fact that PFK-2/FBPase-2 enzymes are induced in hypoxia [37,38,39], an important component of the tumor microenvironment that regulates, for example, tumor angiogenesis and metastasization [18]. It has been suggested that cancer cells may express variable levels of different PFK-2/FBPase-2 enzymes and modulate their relative kinase and/or bisphosphatase activity according to their temporal and spatial metabolic needs [18].

All evidence suggests that PFK-2/FBPase-2 is a potential good target for cancer therapy.

To date, efforts have been focused on inhibition of the PFK-2 activity of PFKFB3 [40].

The choice of this isoenzyme as a target is justified by the fact that its expression is induced by several oncogenes and by hypoxia, as well as by the suggestion that it may contribute to the high glycolytic activity of cancer cells [18].

Recently, it has been shown that PFKFB4 regulates transcriptional reprogramming by enhancing the transcriptional activity of the oncogenic steroid receptor coactivator-3 (SRC-3), deregulation of which is frequently associated with aggressive metastatic tumors [41].

The phosphorylation of SRC-3 at Ser857 by PFKFB4 increases its transcriptional activity and promotes the synthesis of genes that direct the glucose flux towards purine synthesis. This so-called PFKFB4–SRC-3 axis is enriched in oestrogen receptor-positive breast tumors [41].

Additionally, by affecting the SRC3/Akt/mTOR pathway (that regulates autophagy) PFKFB4 functions as a bridge between glycolysis and autophagy. Although autophagy can be involved in both tumor suppression and tumor promotion, it has been proposed that the downregulation of PFKFB4 (or the inhibition of its kinase activity) can help inhibit the SRC3/Akt/mTOR pathway, and hence direct autophagy to promote apoptosis of tumor cells [42].

There is also evidence showing that the FBPase-2 domain of PFKFB4 is important for cancer cell survival, which indicates the phosphatase domain as a new potential therapeutical target for cancer [43]. The selective inhibition of the FBPase-2 activity of the PFKFB4 isoenzyme could ultimately lead to irreversible cellular damage caused by the accumulation of reactive oxygen species as a result of simultaneous high glycolytic flux and depletion of metabolites from the pentose phosphate [18].

The major limitation in the discovery of specific inhibitors of the PFK-2/FBPase-2 phosphatase domains lies in the lack of unique topological features among the different isozymes [18]. The simultaneous inhibition of the FBPase-2 activity of other isozymes, particularly in the liver, impairs the organism’s normal metabolic homeostasis and originates secondary effects [18]. One possible way to overcome this limitation is through the use of computer-aided drug design, a strategy that has been gaining particular relevance in recent years due to the large increase in high-performance computing resources and the development of new in silico approaches [44].

### 2.4. Inhibitors

**Figure 4:**
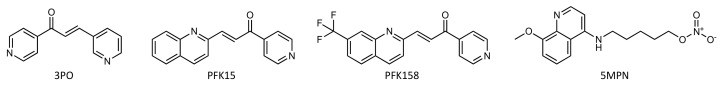
Chemical structure of PFKFB3 (3PO, PFK15 and PFK158) and PFKFB4 (5MPN) inhibitors.

To date, many PFKFB3 inhibitors have been designed, synthesized, and tested in vitro and/or in vivo to evaluate their potential in anti-cancer therapy [45]. Among the different proposed molecules, the charcolones derived from the 3-(3-pyridinyl)-1-(4-pyridinyl)-2-propen-1-one (3PO) molecule (Figure 4) have gained particular attention [46]. The 3PO was the first PFKFB3 inhibitor proposed; however, its poor solubility and selectivity together with the high dose required to achieve potency limited its potential use in clinical trials [47]. To overcome those limitations a large number of 3PO derivatives were synthesized, including PFK15 (Figure 4), which showed increased selectivity and inhibitory effectiveness when compared with 3PO [48]. Further structural optimizations led to PFK158 (Figure 4), a PFK15 derivative that demonstrated a favorable preclinical therapeutic index and superior efficacy and pharmaceutical properties to 3PO and PFK15 both in vitro and in vivo [49]. Its characteristics turned PFK158 into the first-in-man and first-in-class PFKFB3 inhibitor to be evaluated in a phase I clinical trial in patients with advanced solid malignancies (NCT02044861) [50].

Although the great majority of the studies were focused on the inhibition of PFKFB3, there is record of at least one promising PFKFB4 inhibitor, 5-(n-(8-methoxy-4-quinolyl)amino)pentyl nitrate (5MPN) (Figure 4), that demonstrated an anti-proliferative effect of cancer cells both in vitro as in vivo [51]; 5MPN is a PFKFB4 specific inhibitor that binds competitively to its F6P binding site, suppressing kinase activity and consequently reducing the intracellular concentration of F-2,6-BP [51]. Recently, 5MPN was tested in combination therapy with Sunitinib, a receptor tyrosine kinase inhibitor used as a chemotherapeutic agent, showing promising results [52].

## 3. 5-aminoimidazole-4-carboxamide Ribonucleotide Formyltransferase/Inosine Monophosphate Cyclohydrolase (ATIC)

### 3.1. Biological Role

The purine bases are a group of heterocyclic aromatic organic compounds composed of a pyrimidine ring fused to an imidazole molecule. Two of the most common purines are adenine and guanine, which are constituents of vital biomolecules including ATP, GTP, cAMP, DNA, RNA, NADH, FAD, and coenzyme A among others. The purine nucleotides can be obtained by two biosynthetic pathways: the salvage pathway, in which nucleotides are retrieved after the breakdown of nucleic acids or coenzymes, and the de novo biosynthetic pathway, a highly conserved pathway in which phosphoribosyl pyrophosphate (PRPP) is converted into inosine 5′-monophosphate (IMP), the departing nucleotide for the posterior synthesis of AMP and GMP [53].

In humans, the purine requirement for normal cellular growth can be largely maintained through the salvage pathway. However, in the case of rapidly dividing cells, like cancer cells, there is an enormous increase in RNA production and DNA replication. Additionally, the metabolic demand of those cells is so high that they are reprogramed to increase the use of the anabolic pathways. This metabolic change generates elevated purine requirements that can only be supported through the maintenance of the de novo purine synthesis pathway at high levels [54,55].

In humans, the de novo purine biosynthetic pathway requires six enzymes and ten successive steps to convert PRPP into IMP [56]. The 5-aminoimidazole-4-carboxamide ribonucleotide formyltransferase/inosine monophosphate (IMP) cyclohydrolase (ATIC) enzyme is a cytosolic bifunctional enzyme that catalyzes the final two steps of the pathway.

**Figure 5:**
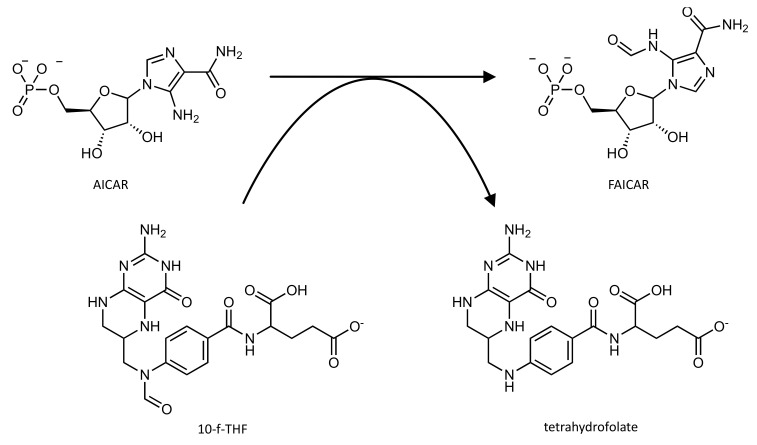
Schematic representation of the reaction catalyzed by the AICAR TFase domain of the ATIC enzyme.

Its AICAR formyltransferase (AICAR TFase) domain (EC 2.1.2.3) catalyzes the transfer of the one-carbon formyl group from the cofactor *N*^10^-formyl-tetrahydrofolate (10-f-THF) to the substrate 5′-phosphoribosyl-5-aminoimidazole-4-carboxamide (AICAR) in order to produce the products 5′-phosphoribosyl-5-formaminoimidazole-4-carboxamide (FAICAR) and tetrahydrofolate (Figure 5).

**Figure 6:**
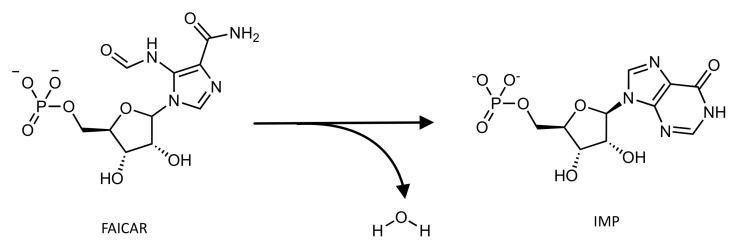
Schematic representation of the reaction catalyzed by the IMPCHase domain of the ATIC enzyme.

Its IMP cyclohydrolase domain (IMPCHase) (E.C. 3.5.4.10) enhances the intramolecular cyclization of the product of the AICAR TFase domain (FAICAR) to the final product of the de novo purine biosynthesis pathway, IMP (Figure 6).

### 3.2. Protein Structure

**Figure 7:**
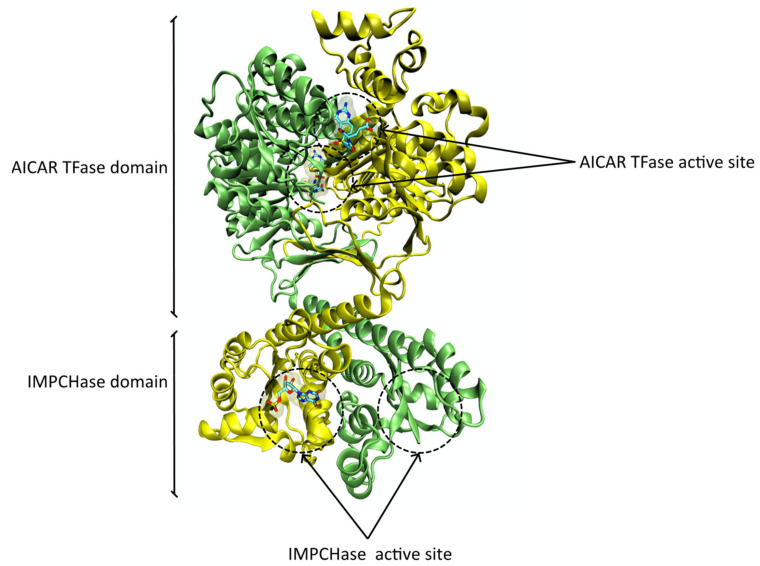
Ribbon representation of the human ATIC enzyme with the PDB ID 1P4R [57] obtained with VMD. The AICAR TFase active site harbors the folate-based inhibitor BW1540U88UD, and the IMPCHase active site (of the ATIC monomer colored in yellow) harbors a xanthosine 5′->monophosphate molecule. All ligands are represented in licorice.

The human ATIC enzyme is an intertwined homodimer with 64 kDa composed by two polypeptide chains with 592 residues each [57]. Each monomer contains a C-terminal AICAR TFase domain composed by residues 199 to 592 and an N-terminal IMPCHase domain composed by residues 1 to 198 [57] (Figure 7). The active sites of each domain are separated by ~50 Å [57] and are not interconnected by any intramolecular tunnel [58].

There is evidence that the human ATIC exists in a monomer/dimer equilibrium [59]. The AICAR TFase active site is located in a long, narrow cleft at the dimer interface where AICAR interacts primarily with one subunit and the folate co-factor with the opposing subunit [60]. Since both subunits are required for AICAR TFase catalytic activity, this domain is only active in the dimer form [59].

The IMPCHase substrate binding site is localized in one monomeric unit away from the dimeric interface, and there is evidence that the IMPCHase domain possesses catalytic activity in both the monomeric and dimeric forms, although the dimeric form is more active [59]. In this case, the obvious advantage of bringing together two different domains in the same enzyme is to place the two catalytic sites in close proximity, avoiding the dilution effects caused by diffusion through solvent. Additionally, there is evidence that the AICAR TFase reaction favors the reverse direction [61]; therefore, the proximity between the two domains raises the high local concentration of FAICAR, favoring its conversion to the final product, IMP, and avoiding its conversion back to AICAR.

### 3.3. Role in Cancer

Attending to the evidence that normal cells preferentially utilize the salvage pathway for the synthesis of purines while tumor cells favor the de novo pathway, it can be expected that the inhibition of enzymes exclusively involved in the de novo pathway represents a good strategy to effectively inhibit cancer growth with minimal damage to normal cells [62]. Although the significance of ATIC in human cancer requires further investigation, there are several studies strongly suggesting that ATIC is a good target against several different cancers [63,64,65]. One such study demonstrated that the inhibition of the AICAR TFase activity of ATIC and the subsequent rise in intracellular levels of 5-Aminoimidazole-4-carboxamide ribonucleotide plays a significant role in the anti-tumorigenic effects of pemetrexed, a drug used in the treatment of non-small cell lung cancer [63,64]. A recent study also showed that ATIC is upregulated in Hepatocellular carcinoma (HCC) tissues, and high levels of ATIC are correlated with poor survival in HCC patients. The inhibition of ATIC expression in cancer cells resulted in a dramatic decrease of cell proliferation and migration and in the increase of cell apoptosis [65].

### 3.4. Inhibitors

**Figure 8:**
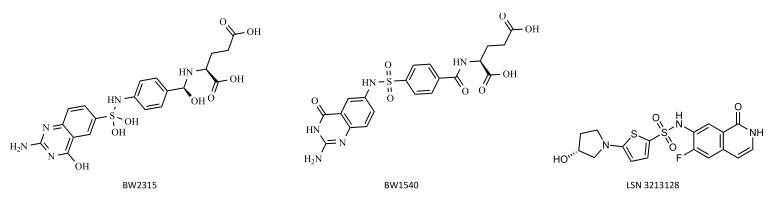
Chemical structure of AICAR TFase inhibitors.

The first ATIC inhibitors developed were two sulfamido-bridged 5,8-dideazafolate analogs, designated BW1540 and BW2315 [57] (Figure 8). They are anti-folate inhibitors specific for the AICAR TFase active site; however, they have never been tested in vitro or in vivo. Recently, a potent new ATIC inhibitor designated LSN3213128 (Figure 8) has been proposed. It is a folate competitive non-classical anti-folate inhibitor selective for AICAR TFase relative to other folate dependent enzymes [66]. In vivo studies have shown that LSN3213128 is orally bioavailable and that it demonstrates anti-tumor activity in murine models [66,67].

To data there are no records of any specific ATIC inhibitors in active clinical development.

## 4. Leukotriene A_4_ Hydrolase (LTA_4_H)

### 4.1. Biological Role

Inflammation is a major pathological characteristic of a wide array of severe endemic illnesses potentially affecting almost all tissues and organ systems of the human body. The development and maintenance of inflammation are governed by a complex network of cellular and soluble factors. Among these are the eicosanoids, a class of structurally related paracrine hormones derived from the metabolism of arachidonic acid, which include the prostaglandins, leukotrienes and lipoxins. During inflammation, the cytosolic enzyme phospholipase A2 releases arachidonic acid from cell membrane phospholipids; the free arachidonic acid can then be converted to prostaglandins by cyclooxygenase or to leukotrienes by the lipoxygenase pathway [68].

The leukotrienes (LTs) are lipid mediators that act during the first phase of inflammation triggered by injury or pathogen invasion [69]. As the name indicates, they are primarily formed in leukocytes and can be divided into two major classes: dihydroxy acid leukotriene B4 (LTB_4_), which is a potent chemotactic agent, and the cysteinyl leukotrienes (CysLTs), which are a group of three (LTC4, LTD4, LTE4) potent spasmogenic agents [69].

The most important leukotriene synthesized during acute inflammatory responses is LTB_4_. It is derived from cell membrane phospholipids by the sequential actions of phospholipase A_2_, 5-lipoxygenase (5-LO) and LTA_4_ hydrolase [68]. It operates its pro-inflammatory functions through the activation of leukocytes and the extension of their survival, acting as a chemoattractant (which induce directed movement of their targets in a concentration-dependent manner) for macrophages and neutrophils, and through the stimulation of leukocyte adhesion to vascular endothelium by the upregulation of integrin expression [68,70]

The chemotactic activity of LTB_4_ is mediated through its binding to two specific G-protein-coupled receptors (GPCR), BLT1 (high affinity and specific for LTB_4_) and BLT2 (non-specific and low affinity for LTB_4_) [71].

Among the panoply of molecules that are supposed to intervene directly or indirectly in the inflammatory process is the tripeptide Pro-Gly-Pro (PGP). It is derived from the extracellular matrix collagen through a multistep proteolytic pathway involving matrix metalloproteases 8 and 9 and the serine protease prolyl endopeptidase [72]. It has been proposed that the PGP peptide functions as a neutrophil chemoattractant by binding to the GPCR chemokine receptors CXCR1/2 [73]. Although there are many studies reporting the presence of significant quantities of PGP in patients with chronic neutrophilic lung diseases (e.g., chronic obstructive pulmonary disease, cystic fibrosis, and bronchiolitis obliterans syndrome) [72,73,74,75,76,77], a recent study did not find evidence of PGP’s role as a chemoattractant of human and mouse neutrophils [78]. Further studies are required to elucidate the effective biological role of PGP.

Interestingly, the levels of these two chemically unrelated molecules (LTB_4_ and PGP) are inversely controlled by the same enzyme, Leukotriene A4 hydrolase (LTA4H). LTA4H is a bifunctional enzyme that has been detected in almost all mammalian cells, organs, and tissues [79]. In a common active center, it catalyzes the final rate limiting step in the biosynthesis of LTB4 through its epoxide hydrolase activity, and catalyzes the hydrolysis of the tripeptide Pro-Gly-Pro (though not the *N*-acetyl PGP) through its aminopeptidase activity [78]

**Figure 9:**
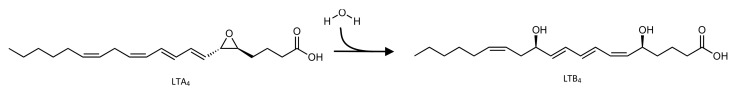
Schematic representation of the reaction catalyzed by the epoxide hydrolase activity of the LTA_4_H enzyme.

The epoxide hydrolase activity of LTA_4_H (EC 3.3.2.6) converts the transient epoxide LTA_4_ (5S-5,6-oxido-7,9-*trans*-11,14-*cis*-eicosatetraenoic acid) into the dihydroxy acid LTB_4_ (5S,12R-dihydroxy-6,14-*cis*-8,10-*trans*-eicosatetraenoic acid) [80,81,82] (Figure 9).

**Figure 10:**
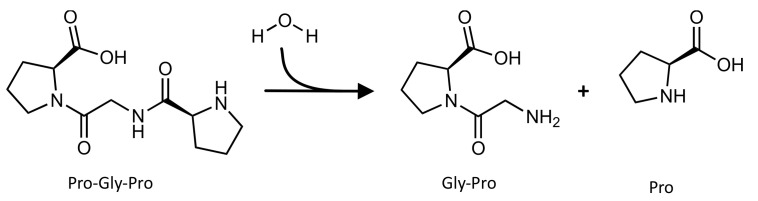
Schematic representation of the reaction catalyzed by the aminopeptidase activity of the LTA_4_H enzyme.

The aminopeptidase activity of LTA4H (EC 3.4.11.6) catalyzes the N-terminal cleavage of the Pro-Gly-Pro tripeptide into the Pro-Gly dipeptide and the amino acid proline [81,82,83] (Figure 10).

Mechanistic proposals show that the residues Glu296 and Tyr383 are specifically required for the aminopeptidase reaction, and Asp375 is only required for the epoxide hydrolase reaction. However, Zn^2+^ Glu-271 and Arg-563 are necessary for both catalytic mechanisms [81,82]. Interestingly, it has been suggested that the LTA_4_H developed from an ancestral aminopeptidase which initially possessed an allosteric lipid binding site, and that the enzyme’s architecture has evolved into an active site capable of accommodating LTA_4_. Subsequent structural optimizations have further improved the substrate alignment, culminating in the establishment of an efficient catalytic mechanism for the conversion of LTA_4_ into LTB_4_ [81].

### 4.2. Protein Structure

**Figure 11:**
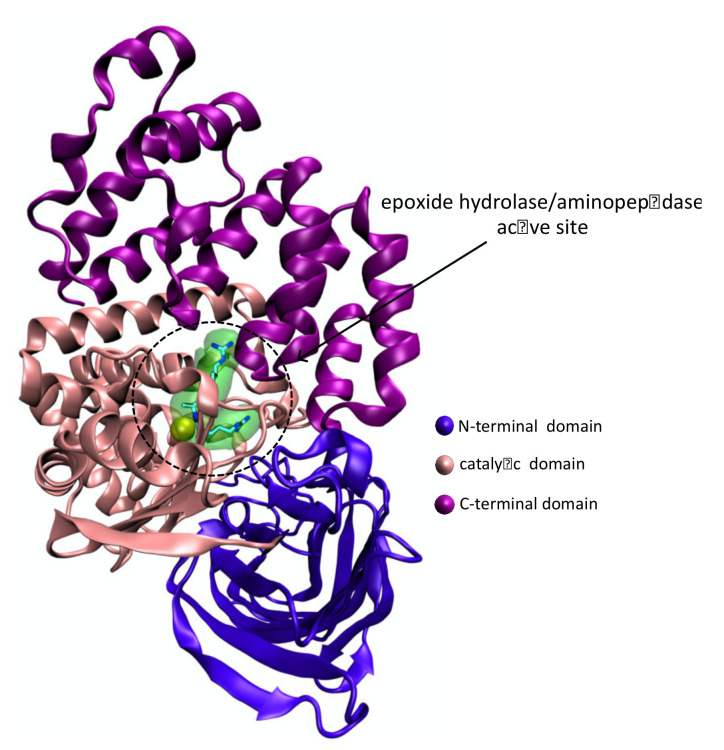
Ribbon representation of the human LTA_4_H enzyme with the PDB ID 3B7T [86] obtained with VMD. The LTA4H active site harbors a catalytic Zn^2+^ ion (colored in yellow) and an Arg-Ala-Arg substrate. All ligands are represented in licorice.

The LTA_4_H is a monomeric 69-kDa cytosolic bifunctional zinc metalloenzyme with 611 amino acids and which is folded into three domains, the N-terminal, catalytic, and C-terminal domains. These domains are packed in a flat triangular arrangement, with an L-shaped cavity between them [84] (Figure 11). The two distinct catalytic activities of LTA_4_H occur in two distinct but overlapping active sites located in that deep L-shaped cavity (Figure 11). One arm of the cavity is wider and highly hydrophilic; it starts near the protein’s surface, where the substrates enter the cleft, then bends and narrows at the site of the catalytic Zn^2+^ into another arm that is predominantly a hydrophobic tunnel that penetrates deeper into the protein [84]. The LTA_4_ occupies the entire cavity, with its epoxide coordinating with the zinc and its long hydrophobic tail buried into the narrow and hydrophobic tunnel [85]. The Pro-Gly-Pro is confined to the wide hydrophilic arm of the cavity that contains the catalytic zinc [85].

### 4.3. Role in Cancer

LTA_4_H is overexpressed in several cancers including colorectal [87], lung and esophageal [88,89], skin squamous cell carcinoma [90], and oral squamous cell carcinoma [91], and several studies have shown that its hydrolase function is implicated in cancer development [87,90,91,92,93,94].

It has been proposed that LTA4H and the product of its epoxide hydrolase activity, LTB_4_, may play an important role in chronic inflammation-associated carcinogenesis via several mechanisms, including the autocrine and paracrine growth-stimulatory effect of LTB_4_ (produced respectively in epithelial cells and inflammatory cells) on precancerous and cancer cells, and the inflammation–augmentation effect on inflammatory cells through positive feedback mediated by its BLT1 receptor and downstream signaling molecules [89].

A recent study has suggested that LTA_4_H is a key modulator of the cell cycle through its negative effect on the expression of the tumor suppressor p27 protein [90]. The Cyclin-dependent kinase inhibitor 1B (CDKN1B, p27^Kip1^), known as p27 protein, controls the transition from the G1 phase into the S phase of the cell cycle [95]. The inactivation of p27 is generally accomplished post-transcriptionally by the oncogenic activation of various pathways that accelerate the proteolysis of the p27 protein and allow cancer cells to undergo rapid division and uncontrolled proliferation. The absence or reduction of p27 protein expression is also reported to be associated with a poor prognosis in several human cancers [95,96,97,98,99,100]. The depletion of LTA_4_H enhances p27 protein stability by mediating the downregulation of its ubiquitination. This ultimately leads to a decrease in cancer cell growth by inducing cell cycle arrest at the G0/G1 phase [90]. Taken together, all of the evidence suggests that inhibiting LTA4H epoxide hydrolase activity is a promising strategy for cancer prevention.

### 4.4. Inhibitors

**Figure 12:**
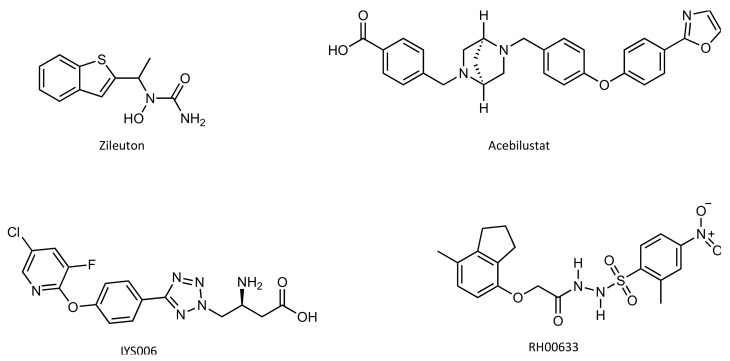
Chemical structure of one 5-LO inhibitor (Zileuton) and three LTA4H inhibitors (Acebilustat, LYS006 and RH00633).

The only compound currently available on the market that interferes with LTB4 biosynthesis is Zileuton (Figure 12), a 5-LO inhibitor and a very weak inhibitor of LTB_4_ biosynthesis [101]. It has only been approved in the United States for the treatment of asthma, and has some disadvantages, for example dose-limiting toxicity and unfavorable pharmacokinetic properties [102].

The development of an inhibitor specifically targeting LTA4H would be advantageous because it would allow the inhibition of LTB4 synthesis without affecting the biosynthesis of other lipids that depend on the upstream enzymes (e.g., 5-LO) [103]. Researchers and the pharmaceutical industry have been actively searching for selective and potent LTA4H inhibitors for over ten years (see [103] for review). During this time, several inhibitors of LTA4H have been proposed, and five of those molecules have reached the early clinical development stage, although none of the clinical trials has targeted cancer patients [102]. Of these, only two, Acebilustat from Celtaxsys [104] and LYS006 from Novartis [105] (Figure 12), remain in active clinical development. Acebilustat completed Phase 2 clinical trials for cystic fibrosis (NCT02443688 [106]) and for Acne Vulgaris (NCT02385760 [107]). LYS006 is in ongoing Phase 2 clinical trials for hidradenitis suppurativa (NCT03827798 [108]), inflammatory acne (NCT03497897 [109]), ulcerative colitis (NCT04074590 [110]), nonalcoholic steatohepatitis and non-alcoholic Fatty Liver Disease (NCT04147195 [111]).

All five proposed molecules inhibit both epoxide hydrolase and aminopeptidase catalytic activities of the enzyme [102].

Recently, a research group has used computer-aided drug design to search for new effective and selective LTA_4_H inhibitors. In the last stage of the study, they experimentally evaluated the epoxide hydrolase inhibitory activities of the five best scored hits found in silico. Among the tested compounds, the one designated RH00633 (Figure 12) stands out with 73.6% inhibition of the basal epoxide hydrolase activity of LTA_4_H. RH00633 binds to the enzyme’s active site and interacts with the catalytic Zn2+, along with several other important catalytic residues [112].

Several studies have suggested an association between the difficulties in the discovery of a potent and selective LTA4H and the simultaneous inhibition of both catalytic activities of the enzyme [113,114]. These studies suggested that a good LTA4H inhibitor should be epoxide hydrolase selective and aminopeptidase sparing, in order to reduce the production of LTB4 while retaining the ability to reduce PGP levels.

The fact that the Pro-Gly-Pro binding site is confined to the wide hydrophilic arm of the cavity while the LTA_4_ occupies the entire cavity suggests that the selective blockade of the hydrophobic tunnel where the long hydrophobic tail of LTA_4_ is buried without interfering with the aminopeptidase active site would be a good strategy for the selective inhibition of the epoxide hydrolase activity of LTA4H [83,85]. However, all the aminopeptidase-sparing LTA4H inhibitors proposed to date have shown very low potency in inhibiting the epoxide hydrolase activity of LTA4H when compared to general LTA4H inhibitors [78,85]. Because there are no solid data to date about the true physiological role of PGP, the physiological relevance of sparing the aminopeptidase function of LTA4H remains questionable [102].

## 5. Jumonji Domain-Containing Protein 6 (Jmjd6)

### 5.1. Biological Role

Posttranslational modifications (PTMs) are a variety of covalent processing events that change the properties of a protein through proteolytic cleavage or the addition of a modifying group, such as hydroxyl, acetyl, phosphoryl, glycosyl, methyl, etc., to one or more amino acids [115]. To date, more than 400 different PTM have been identified [116]. They can be reversible or irreversible, and inevitably affect the structure and the dynamics of the proteins that play key roles in a panoply of biological processes [116,117,118].

Protein hydroxylation is a reversible post-translational modification that occurs with more frequency in proline, and can occur in lysine, asparagine, aspartate or histidine as well, among other amino acids [119]. Although for a long time it was mainly considered a specialized post-translational modification of the extracellular collagens and proteins with collagen-like sequences, the recent discovery of new hydroxylation substrates demonstrates that protein hydroxylation can extensively influence cell signaling pathways. In general, protein hydroxylation can modify protein stability, affect the enzymatic activity of certain proteins through the perturbation of their interaction with direct activators, and influence the occurrence of other post-translational modifications that affect their activity in turn [119].

Methylation is one of the most common post-translational modifications, and has been implicated in the regulation of transcription [120], signal transduction [121], nuclear transport [122], T-cell activation [123], protein trafficking, and protein repair [124] among many other cellular processes [125]. It usually occurs at the nitrogen atom of arginine or lysine side chains or at the terminal α-amino group of polypeptides.

About 0.5% of all arginine residues in mammalian tissues are methylated [126]. Arginine is the most basic of all amino acids, with a p*K*_a_ of ~13.8. It contains a guanidinium group that is protonated at physiological pH, generating a positive charge that is very important for the establishment of several intramolecular and intermolecular interactions, including hydrogen bonds (it has five hydrogen donors) and cation-pi interactions with aromatic rings or salt bridges (it can mediate the formation of two salt bridges simultaneously).

The methylation of the guanidinium group of arginine delocalizes its positive charge, raising its hydrophobicity and consequently increasing its affinity to aromatic rings in cation–pi interactions [127]. It removes one potential hydrogen bond for each methyl group added, and it increases its side chain bulkiness [126,128]. These chemical changes of the arginine side chain regulate its binding to certain protein domains that are “readers” of methylarginine marks (e.g., Tudor domains) [126]. Ultimately, arginine methylation can both positively and negatively regulate protein–protein interactions [125].

Posttranslational arginine methylation occurs in hundreds of proteins, usually affecting protein-protein interaction or protein stability [129]. In the case of histones, the methylation of arginine plays an important role in the epigenetic regulation of gene expression by altering chromatin structure [125]. There are three different types of arginine methylation that occur in mammalian cells, monomethylarginine, asymmetric dimethylarginine, and symmetric dimethylarginine, and they are all catalyzed by a family of nine protein arginine methyltransferases [126].

Although there is evidence that arginine methylation is reversible, to date only one enzyme, Fe(II) and 2-oxoglutarate-dependent dioxygenase Jumonji domain-containing protein 6 (Jmjd6) (EC 1.14.11.-), has been reported to have potential arginine demethylation activity in vivo [130,131].

Jmjd6 is a bifunctional enzyme that also catalyzes the hydroxylation of lysine in a wide variety of target molecules [131,132,133].

Both the demethylation and hydroxylation mechanisms catalyzed by Jmjd6 require the presence of Fe (II) as a cofactor and 2-oxoglutarate (2-OG) as a co-substrate [134,135].

**Figure 13:**
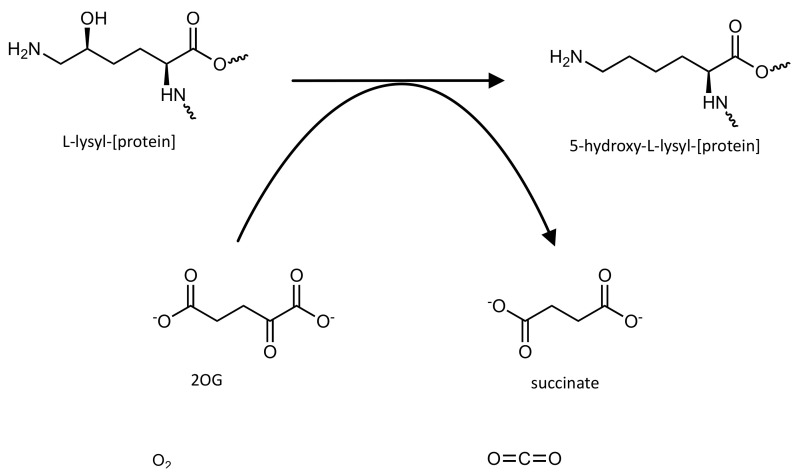
Schematic representation of the reaction catalyzed by the lysine hydroxylase activity of the Jmjd6 enzyme.

The hydroxylation mechanism starts with the sequential binding of 2OG, the substrate and dioxygen, to the active site containing a Fe (II) ion. Then, the oxidative decarboxylation of 2OG results in the carbon dioxide, succinate and a ferryl intermediate that mediate substrate oxidation. Hydroxylation can occur via a radical rebound mechanism or via direct insertion of oxygen from the ferryl intermediate into the requisite C–H bond [135] (Figure 13).

**Figure 14:**
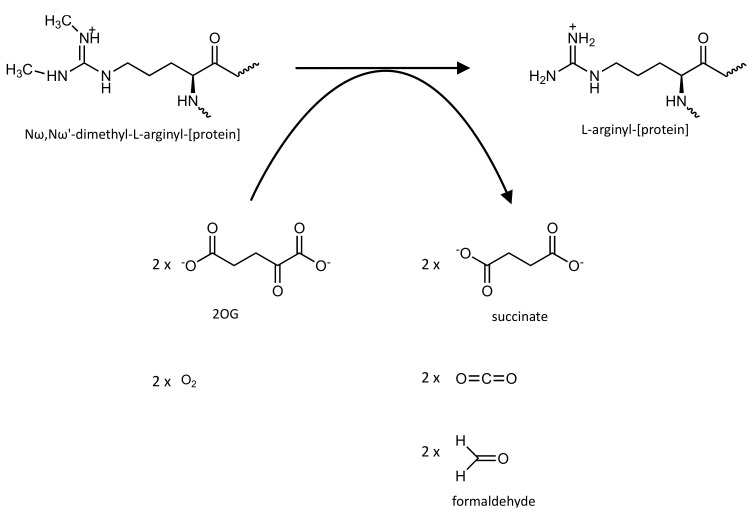
Schematic representation of the reaction catalyzed by the arginine demethylation activity of the Jmjd6 enzyme.

The demethylation catalytic mechanism is thought to be preceded by a hydroxylation reaction that produces an unstable hydroxymethyl-lysine intermediate, succinate, and CO_2_. The hydroxymethyl group of the hydroxymethyl–lysine intermediate is then spontaneously lost as formaldehyde, releasing the methyl group and producing an unmodified arginine residue [134] (Figure 14).

Recent studies suggest that in addition to its demethylase and hydroxylase activities, this enzyme may also have a kinase [136] and a protease function [137].

### 5.2. Protein Structure

**Figure 15:**
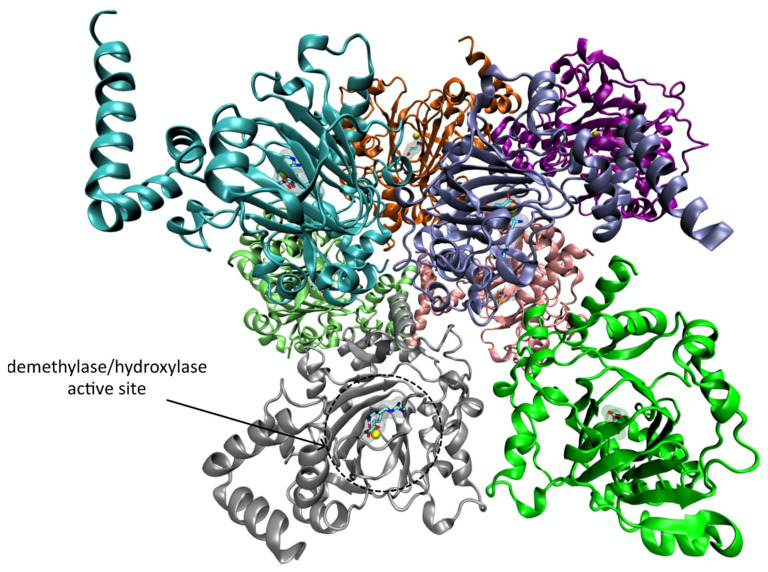
Ribbon representation of the human Jmjd6 enzyme with the PDB ID 6MEV [137] obtained with VMD. The Jmjd6 active site harbors a catalytic Fe (II) ion (colored in yellow), one molecule of mono-Methyl Arginine and one molecule of 2-oxoglutaric acid. All ligands are represented in licorice.

JMJD6 is a 47.5 kDa protein with 403 amino acids that belongs to a family of Jumonji domain (JmjC)-containing proteins which are non-haeme iron (II) and 2-oxoglutarate (2OG or α-ketoglutarate)-dependent oxygenases. JMJD6 can exist as a monomer, but in solution adopts an oligomeric form, which can be a trimeric, pentameric or larger oligomeric form [138] (Figure 15), organized in ring like structures that upon deletion of it poly-Ser sequence turn into a fibril form [119,139].

Its structure comprises a double-stranded β-helix (DSBH) fold, characteristic of the 2OG-dependent oxygenases, which is surrounded by characteristic secondary structure elements [139]. The Fe(II) binding site of the catalytic center is located in the opening end of the barrel-like structure formed by the strands β1 and β2 of the typical DSBH fold (Figure 15). The Fe(II) is coordinated with the side chain of His187, Asp189 and His273, which form a conserved HXD/E(X)nH motif that is essential for the enzyme’s catalytic activity [119,139,140]. Structural analysis of JMJD6 shows that in addition to its Jumonji domain, it also possesses other motifs, namely a DNA binding motif (AT-hook), five nuclear localization signals, a nuclear export signal, a SUMOylating site, and a C-terminal polyserine (poly-Ser) domain (residues 340–359) that is involved in regulating its oligomerization and cellular localization [119,138,139].

### 5.3. Role in Cancer

Epigenetics is a term used to describe heritable changes in gene expression without alteration in DNA sequences. The key processes responsible for epigenetic regulation include DNA methylation, histone modification (e.g., methylation, hydroxylation, acetylation, ubiquitination, etc.) nucleosome remodeling, and alterations in non-coding RNA profiles [141]. The deregulation of the epigenetic processes leads to altered gene functions and ultimately to wide variety of pathologies including cancer, metabolic diseases, autoimmune diseases and neurological disorders, among others [138]. The activities of JMJD6 as arginine demethylase [130], lysyl hydroxylase [142] and eventually as tyrosine kinase [136] of histones suggests that this enzyme may have an important role in the epigenetic regulation of chromosomal rearrangement and gene transcription.

The involvement of JMJD6 in many developmental processes including embryogenesis [143], angiogenesis [144] and tumorigenesis has been demonstrated [145]. Recently, Yang and colleagues compilated a series of studies relating the abnormal overexpression of JMJD6 in several different cancers (e.g., Breast cancer, Melanoma, Oral cancer, Glioblastoma, Hepatocellular carcinoma, Colon carcinoma, Ovarian cancer, Neuroglioma, etc.) to increased cancer cell proliferation and invasion leading to aggressive tumors and poor prognosis [138].

Both the histone arginine demethylase and lysyl hydroxylase activities of JMJD6 have been associated with tumorigenesis. In glioblastoma and neuroblastoma, JMJD6 upregulates target gene transcription by forming a complex with Bromodomain-containing protein 4 (BRD4)—a transcriptional and epigenetic regulator associated with cell cycle control—and demethylating the histone H4 at arginine 3 (H4R3) target gene antipause enhancers, leading to RNA polymerase II release from promoter-proximal pause regions and consequently to aberrant gene expression [146,147,148]. In colon carcinoma, JMJD6 complexes with the tumor suppressor protein p53 and catalyzes its hydroxylation, resulting in the repression of its transcriptional activity. It has been demonstrated that the knockdown of JMJD6 represses p53-dependent cell proliferation and tumorigenesis in vivo [149].

Overall, the existing evidence suggests that the simultaneous inhibition of both the demethylase and hydroxylase activities of JMJD6 may be a promising strategy for effective cancer therapy.

### 5.4. Inhibitors

**Figure 16:**
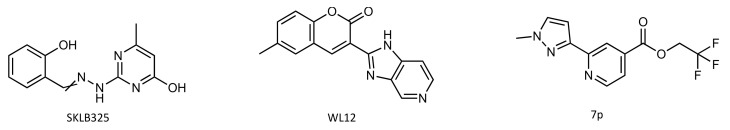
Chemical structure of the JMJD6 inhibitors.

To date, only three molecules have been proposed as drug candidates targeting JMJD6, and none of them has reached an early clinical development stage. SKLB325 (Figure 16) was designed, synthesized, and tested to evaluate its antitumor activity against ovarian cancer cells in vivo and in vitro [150]. The results demonstrated that it suppresses ovarian cancer growth through inhibition of proliferation and induction of apoptosis and cell death. In vivo tests demonstrated that the administration of SKLB325 to tumor-bearing mice prolonged survival without obvious side effects [150].

WL12 (Figure 16) was designed to bind to the 2OG-binding site of JMJD6 and inhibit its demethylase activity. It was tested in vitro and demonstrated the ability to suppress the JMJD6-dependent proliferation of cervical and liver cancer cells [151]. A new potent and selective JMJD6 inhibitor, 7p (Figure 16), was recently proposed; however, it still requires in vitro and in vivo validation [152].

## 6. Current and Future Developments

The four enzymes described here possess different biological functions and different structural and functional characteristics; however, they all share two characteristics: they are promising drug targets against cancer, and they are multifunctional.

Multifunctionality is an advantage that must be exploited for the development of new potent and selective inhibitors. Depending on an enzyme’s particular features, such as the number and location of active sites, possession or not of allosteric regulation, provenience of the substrates, etc., its catalytic activity can be inhibited using multiple approaches.

In the case of the PFK-2/FBPase-2 enzyme, there is evidence of the anti-cancer benefits accomplished by the inhibition of both its kinase [45] and phosphatase activities [18]. Considering that the PFK-2 domain is only active in the dimer form, there are three different strategies that can be used to target this enzyme for anti-cancer treatment: (1) selective inhibition of PFK-2 activity; (2) selective inhibition of FBPase-2 activity; and (3) the inhibition of PFK-2 activity by preventing monomeric dimerization.

In the case of ATIC, the product of the IMPCHase domain is the substrate of the AICAR Tfase domain. Hence, the inhibition of the former prevents the activity of the latter through the elimination of its substrate. Additionally, there is also evidence that AICAR TFase catalytic activity is only active in the dimeric form [59]. Because both catalytic activities are essential for purine biosynthesis in cancer cells, three different strategies can be used to target ATIC for anti-cancer treatment: (1) selective inhibition of IMPCHase activity; (2) selective inhibition of AICAR TFase activity; and (3) the inhibition of AICAR TFase activity by preventing monomeric dimerization.

LTA_4_H catalyzes two distinct catalytic activities that occur in two distinct but overlapping active sites. This means that both activities will be inhibited independently of the transition state or substrate analog used to selectively inhibit the enzyme. Although only the inhibition of LTA_4_H epoxide hydrolase activity has been related to anti-cancer effects, there is no evidence that the inhibition of aminopeptidase would bring any beneficial effects; therefore, three strategies can be used to inhibit this enzyme: (1) selective inhibition of epoxide hydrolase; (2) selective inhibition of aminopeptidase; and (3) aminopeptidase-sparing LTA_4_H inhibitors that bind to the hydrophobic tunnel where the long hydrophobic tail of LTA_4_ is buried.

In the case of the Jmjd6, both demethylation and hydroxylation mechanisms catalyzed by Jmjd6 require the presence of Fe (II) as a cofactor and 2-oxoglutarate (2-OG) as a co-substrate, which means that both mechanisms occur in the same active site. Since both histone arginine demethylase and lysyl hydroxylase activities of JMJD6 has been associated with tumorigenesis it is possible to target the enzyme by using either: (1) arginine demethylase selective inhibitors; or (2) lysyl hydroxylase selective inhibitors.

The current status of the development of inhibitors targeting the four enzymes described in this review is summarized in Table 1.

## 7. Conclusions

Multifunctionality is an extraordinary capability restricted to a small number of enzymes. PFK-2/FBPase-2, ATIC, LTA_4_H and Jmjd6 are four multifunctional enzymes with a proven relevant role in the proliferation and/or survival of cancer cells, and their inhibition can increase the life expectancy of some cancer patients.

Although there are studies reporting the identification of potential inhibitors targeting each of the four described enzymes, to date only LTA_4_H and PFK-2/FBPase-2 have inhibitors in active clinical development, and only the PFK-2/FBPase-2 inhibitor (PFK158) is being tested in cancer patients. However, all evidence points to these four enzymes as promising targets for the development of new anti-cancer drugs, and it is our belief that these enzymes’ extraordinary capability to perform different catalytic reactions could be used as an advantage in the development of efficient new molecular-targeted therapies against cancer.

## Figures and Tables

**Table 1:** Current status of the development of inhibitors targeting PFK-2/FBPase-2, ATIC, LTA_4_H and Jmjd6.

Enzyme	Specific Target	Inhibitors	Evaluated in vitro	Evaluated in vivo	Clinical Trials in Cancer Patients
PFK-2/FBPase-2	PFK-2 activity of PFKB3	3PO [46]	yes [46]	yes [46]	no
		PFK15 [48]	yes [48]	yes [48]	no
		PFK158 [48]	yes [49]	yes [49]	NCT02044861 [50]
	PFK-2 activity of PFKFB4	5MPN [51]	yes [51]	yes [51]	no
ATIC	AICAR TFase	BW1540 [57]	no	no	no
	AICAR TFase	BW2315 [57]	no	no	no
	AICAR TFase	LSN3213128 [66]	yes [66,67]	yes [66,67]	no
LTA_4_H	epoxide hydrolase and aminopeptidase activities	Acebilustat	no available data	no available data	no ^(1)^
	epoxide hydrolase and aminopeptidase	LYS006 [105]	yes [105]	yes [105]	no ^(1)^
	epoxide hydrolase	RH00633 [112]	yes [112]	no	no
Jmjd6	Demethylase and hydroxylase	SKLB325 [150]	yes [150]	yes [150]	no
	Demethylase and hydroxylase	WL12 [151]	yes [151]	no	no
	Demethylase and hydroxylase	7p [152]	no	no	no

^(1)^ Currently in Phase 2 clinical trials for other inflammatory conditions.
